# Variation in Subtypes of Obsessive-Compulsive Traits in Migraine Patients Undergoing Onabotulinum Toxin A Therapy

**DOI:** 10.3390/toxins17040199

**Published:** 2025-04-14

**Authors:** Giovanna Viticchi, Lorenzo Falsetti, Chiara Di Felice, Gioacchino De Vanna, Sergio Salvemini, Marco Bartolini, Gianluca Moroncini, Mauro Silvestrini

**Affiliations:** 1 Neurological Clinic, Experimental and Clinical Medicine Department, Marche Polytechnic University, 60120 Ancona, Italygioacchino.devanna@ospedaliriuniti.marche.it (G.D.V.); sergio.salvemini@ospedaliriuniti.marche.it (S.S.); m.bartolini@staff.univpm.it (M.B.); m.silvestrini@staff.univpm.it (M.S.); 2 Clinica Medica, Clinical and Molecular Sciences Department, Marche Polytechnic University, 60120 Ancona, Italy; l.falsetti@staff.univpm.it (L.F.); g.moroncini@staff.univpm.it (G.M.)

**Keywords:** chronic migraine, obsessive-compulsive disorder, onabotulinum toxin A, medication overuse headache, chronic pain

## Abstract

Background: Patients with chronic migraine (CM) associated with medication overuse headache (MOH) often exhibit concomitant psychiatric traits including obsessive-compulsive disorder (OCD). Limited data exist on the impact of migraine therapies on these traits. This study aimed to analyse the influence of onabotulinum toxin A (OBT-A) on OCD in CM + MOH patients. Methods: All CM + MOH patients attending the AOU-Marche Headache Centre and treated with OBT-A over a 9-month period were prospectively analysed. At baseline and every three months, patients completed several questionnaires, including the Obsessive-Compulsive Inventory-Revised (OCI-R), to assess the presence of OCD and its subscales. Results: Thirty patients were enrolled. Repeated measures tests revealed a statistically significant decrease from T0 to T3 in the OCI-R score (*p* = 0.017) and among the different subscales, specifically the checking score (*p* = 0.029). The MIDAS (migraine disability assessment score) and HIT-6 (headache impact test) scores exhibited a statistically significant reduction from T0 to T3 (*p* < 0.0001), similar to the decrease in monthly migraine days and symptomatic medication intake. Conclusions: Patients treated with OBT-A showed significant improvement in OCD, particularly in subscales assessing somatic and aggressive obsessions as well as control compulsions. Several patients transitioned from a CM + MOH condition to an episodic form without drug abuse. The potential impact of OBT-A on psychiatric symptoms warrants further consideration to improve patient management strategies.

## 1. Introduction

Patients suffering from chronic migraine (CM) frequently exhibit associations with psychiatric characteristics that may impact the clinical manifestation of the condition headache. Numerous studies indicate that depression and anxiety are typically linked to CM, particularly when medication overuse headache (MOH) is present [1,2]. They are present in migraine patients two to ten times more often than in the general population [3]. The narrow relationship between depression, anxiety, and migraine is probably multidimensional, with both genetic and pathophysiologic involvement. In fact, low levels of serotonin and dopamine levels have been found in all of these conditions [4,5]. Psychiatric traits are recognised as risk factors for the transition from episodic to chronic migraine [6,7], especially when complicated by MOH [8]. Additionally, these factors significantly affect the quality of life and treatment response [9]. Various psychiatric disorders may be associated with migraines, albeit less frequently. Patients with bipolar disorder often report migraine symptoms, particularly in families with high heritability and significant severity of the psychiatric illness [4].

Fewer data are available on the relationship between CM and obsessive-compulsive disorder (OCD). A few previous studies showed a significant proportion of obsessive-compulsive (OC) traits in patients with CM, ranging from 25 to 36 per cent [10,11], especially when MOH was associated [11,12]. The risk of the underdiagnosis and undertreatment of these patients in daily practice is relevant [13]. Other studies have instead underlined a correlation between OCD and migraine only in the daily headache group [14].

In a previous study, we analysed changes in OC traits in patients with CM + MOH undergoing onabotulinum toxin A (OBT-A) therapy [15]. To assess our patients, we used the validated Italian version of the Obsessive-Compulsive Inventory-Revised (OCI-R) scale [16]. The OCI-R is a self-report questionnaire to assess the severity and type of symptoms consistent with obsessive-compulsive disorder. This scale includes 18 questions corresponding to six subscales (“washing”, “controlling”, “ordering”, “obsessing”, “hoarding”, and “neutralizing”). We found a positive effect of treatment with OBT-A on both migraine severity and globally on OCD traits, also considering a possible effect of OBT-A on these psychiatric aspects of personality.

The present is a pilot study aiming to provide further information on the relevance of obsessive-compulsive traits in patients with chronic migraines and analysed individual subscales of the OCI-R in a new sample of patients undergoing OBT-A therapy, followed prospectively for nine months.

## 2. Results

For this preliminary study, we gathered a cohort of 30 consecutive patients at time point T0. Table 1 summarizes the cohort characteristics at the study entry and the characteristics of the subjects at T1, T2, and T3.

**Figure 1:**
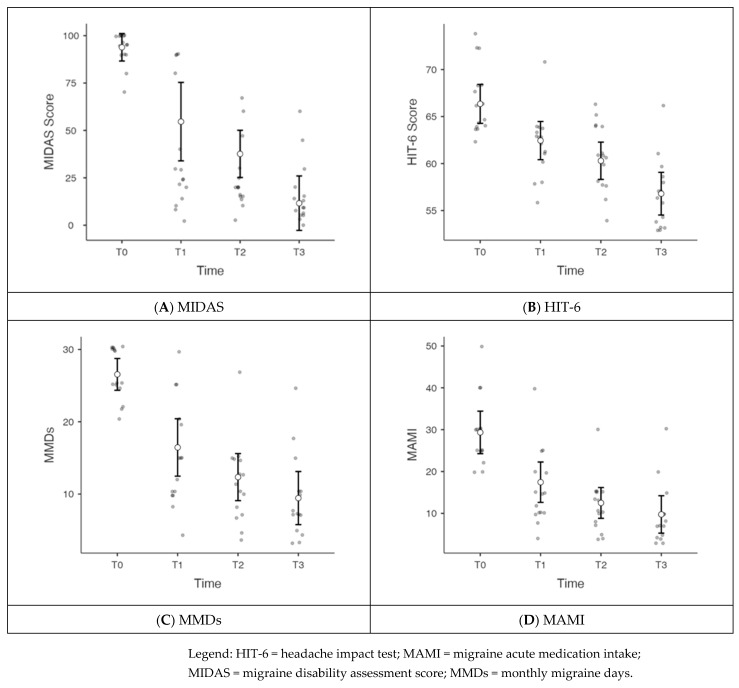
Mean variation of different scores at the four time points (T0 to T3). The figures show the variation in the principal parameters of migraine burden and severity between different time points.

Adopting the ANOVA test for repeated measures, we observed that the MIDAS (migraine disability assessment score) showed a statistically significant reduction from T0 to T3, with a significant within-subject effect (*p* < 0.0001) that remained significant, also correcting for age and sex (*p* = 0.032). The results of the uncorrected model are shown in Table 2 and Figure 1A.

**Figure 2:**
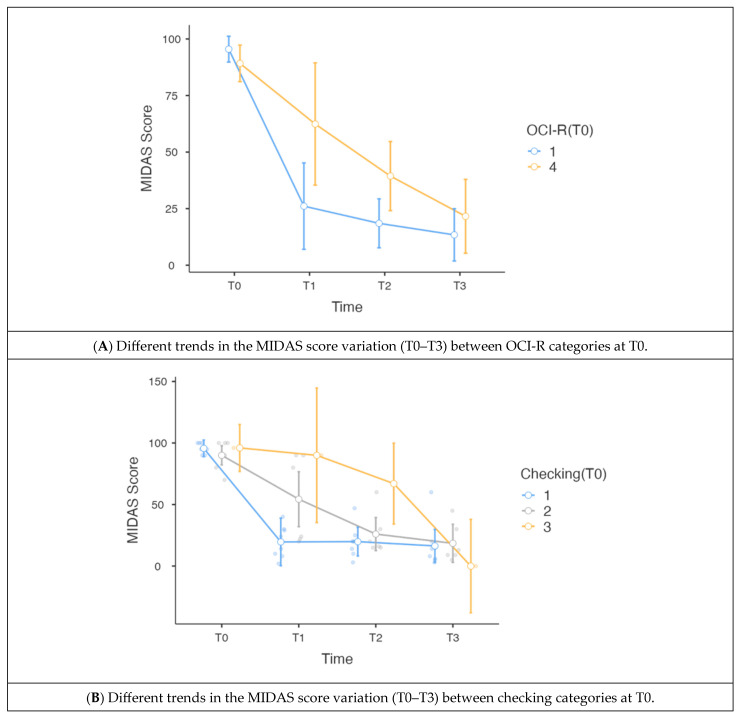
Variation in the MIDAS scores over time by the OCI-R or checking categories. (**A**) OCI-R category 1 exhibited a more rapid improvement in MIDAS scores compared with category 4; (**B**) checking categories 1 and 2 demonstrated a more rapid improvement in the MIDAS scores compared with category 3. Legend: MIDAS = migraine disability assessment score; OCI-R = Obsessive-Compulsive Inventory-Revised.

When we added the OCI-R category at T0 as an independent variable to the uncorrected model, we observed a discriminatory ability and different MIDAS performance between patients with an OCI-R of 1 and those with an OCI-R of 4, as shown in Table 3 and Figure 2A. The full-factorial ANOVA model showed a statistically significant interaction effect between the OCI-R category at T0 and the MIDAS score at the 4 time points in the within-subject effects (*p* = 0.014). The OCI-R category at T0 also resulted in a significant between-subject effect (*p* = 0.040). When correcting this model for age and sex, the OCI-R category at T0 maintained a significant within-subject (*p* = 0.015) and between-subject (*p* = 0.023) effect.

The same full-factorial ANOVA model showed a statistically significant within-subject effect in the interaction of the checking category at T0 with the MIDAS score at the 4 time points (*p* < 0.001), as shown in Table 4 and Figure 2B. The between-subject effect did not present a significant result (*p* = 0.134). The within-subject effect also remained significant (*p* = 0.001) when correcting for age, while correction for sex was not possible due to the data distribution. The between-subject effect did not result in being significant, even after age correction (*p* = 0.157).

Adopting the ANOVA test for repeated measures, we observed that the HIT-6 (headache impact test) score showed a statistically significant reduction from T0 to T3, with a significant within-subject effect (*p* < 0.0001), as shown in Figure 1B. This within-subject effect remained significant after correction for age and sex (*p* < 0.0001). The OCI-R was not associated with different trends in HIT-6 at the four time points (*p* = 0.191) when considered as a between-factors variable in this model.

We also observed that MMDs showed a statistically significant reduction from T0 to T3, with a significant within-subject effect (*p* < 0.001), as shown in Figure 1C. This within-subject effect remained significant even after correction for age and sex (*p* = 0.006). The OCI-R was not associated with different trends at the four time points (*p* = 0.098) when considered as a between-factors variable in this model.

The ANOVA for repeated samples showed that MAMI was statistically reduced from T0 to T3, with a significant within-subject effect (*p* < 0.001), as shown in Figure 1D. This within-subject effect remained significant even after correction for age and sex (*p* < 0.001). The OCI-R was not associated with different trends at the four time points (*p* = 0.0915) when considered as a between-factors variable in this model.

**Figure 3:**
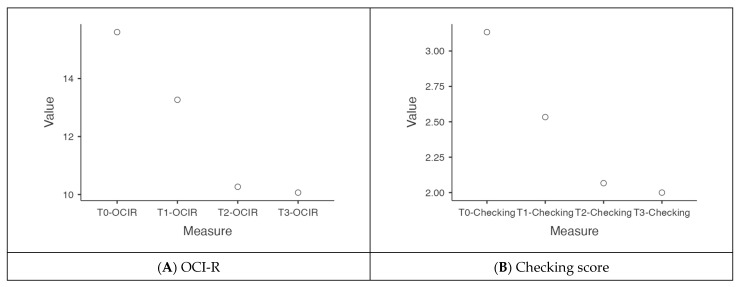
Means of OCI-R and checking at the different time points (T0 to T3). (**A**) The mean of the OCI-R score tended to reduce progressively along the OBT-A therapy visits. (**B**) The mean of the checking score tended to reduce progressively along the OBT-A therapy visits. Legend: OCI-R = Obsessive-Compulsive Inventory-Revised.

OCI-R was found to be statistically reduced from T0 to T3 with a significant within-subject effect (*p* = 0.0419) in the ANOVA test for repeated measures. This observation was confirmed with Friedman’s nonparametric test, which showed a statistically significant decrease in the OCI-R score from T0 to T3 (*p* = 0.017, Figure 3A) and in the OCI-R score percentiles from T0 to T3 (*p* = 0.023), as shown in Table 1.

Friedman’s nonparametric test showed a statistically significant decrease in the overall checking score from T0 to T3 (*p* = 0.029, Figure 3B) and in the percentiles of the checking score from T0 to T3 (*p* = 0.032), as synthesized in Table 1.

Friedman’s test showed no statistically significant changes in global ordering (*p* = 0.321) and global ordering treated in percentiles (*p* = 0.206), global washing (*p* = 0.145) and global washing treated in percentiles (*p* = 0.145), global obsessing (*p* = 0. 256) and percentile-treated global obsessing (*p* = 0.228), global mental neutralizing (*p* = 0.348) and percentile-treated mental neutralizing (*p* = 0.569), and global hoarding (*p* = 0.147) and percentile-treated global hoarding (*p* = 0.176).

## 3. Discussion

The results from this preliminary study confirm the positive effects of OBT-A on both the frequency and severity of migraine attacks as well as on the OC traits. During injection visits, there was a significant reduction in the MMDs, and both the MIDAS and the HIT-6 scores showed a similar trend. Additionally, a significant number of patients exhibited improvements in their OC disease classification. The presence of OC traits in the entire sample was noteworthy, with 26.7 percent of subjects displaying a frankly pathological profile at the baseline.

There are no specific hypotheses in the literature regarding the correlation between migraine and OCD. One possible explanation could be a shared serotonergic dysfunction: migraine patients typically present with low serotonergic levels, and migraine attacks are often triggered by the activation of downregulated receptors [12,17]. Additionally, OCD patients also appear to have low serotonergic levels. PET studies have shown a significant reduction in serotonin transporter availability, which correlates with a high presence of clinical symptoms in OCD subjects [18,19]. Recently, OBT-A has been employed to treat depressive states, social anxiety, and bipolar disorders [20]. Some studies have suggested a possible role for OBT-A in increasing serotonin in various brain areas [21,22,23]. This could be a potential pathophysiological explanation for the improvement observed in both migraine and OCD symptoms in our sample.

Several studies have shown that the presence of comorbidities, particularly psychiatric traits, is a significant risk factor for the chronicisation of migraines and subsequent MOH [1,2,24]. Our patients experienced chronic migraines with MOH, and several studies have indicated that MOH can be viewed as an “abusive behaviour” towards various classes of drugs [25,26]. Furthermore, MOH also serves as a notable risk factor for migraine chronicisation [13].

Previous studies have explored the potential influence of personality traits on clinical responses to OBT-A therapy. For instance, several studies have indicated that migraine patients display a high level of neuroticism. This trait appears to forecast a poor response to both prophylactic and acute treatments [27,28]. In studies of childhood abuse, neuroticism appears to be the strongest predictor of migraine worsening, and is probably the mediator between childhood abuse and migraine [27]. Other studies have shown that personality traits, particularly borderline personality and neuroticism, appear to be the best predictors of a negative response to therapy in migraine, with an increased risk of medication overuse [28]. In our study, the presence of OCD did not counteract the effect of OBT-A, with improvement in a significant proportion of patients.

A recent study showed that dependent personality traits and the borderline emotional instability subtype are significantly associated with non-response to OBT-A in patients with CM [29]. There is evidence that personality traits associated with chronic pain could increase the risk of treatment failure, especially if medication overuse is present [30].

A few studies have explored the presence and impact of obsessive-compulsive disorder in migraine patients. Presumably, most OC traits have remained largely underdiagnosed and are thus often undertreated, with significant consequences on response to treatment and quality of life [13]. Indeed, OC personality traits, whether subclinical or clinically evident, have a negative impact on response to migraine prophylaxis [31]. Moreover, newer therapies, such as anti-CGRP monoclonal antibodies, seem less effective in this patient population [32]. Bottiroli et al. found that the presence of personality disorders belonging to cluster C and obsessive- compulsive personality disorder predicted the failure of erenumab therapy [30]. In our sample, however, patients with OC traits showed a good response to OBT-A, with a significant reduction in both MMDs and MAMI.

During treatment with OBT-A, patients presented a progressive improvement in global OCI-R scores, as already demonstrated in our previous study [15]. In this study, we deepened our analysis. We also found that among the subscales of the OCI-R, the so-called “checking” presented a significant reduction from patient enrolment (T0) until the third session of OBT-A. “Checking” is a subscale that investigates somatic and aggressive obsessions and checking compulsions [33]. It represents, along with “washing”, the most common compulsion among the different aspects of obsessive-compulsive disorder [34], and could be present in 63 percent of affected patients [35]. In our study, we delved into the “checking” item in its subscales, showing that all subscales showed significant improvement over the visit during the treatment period. However, looking at the time course (Figure 2B), it is evident that patients with sub-item 1 (the mildest form) presented a rapid, significant improvement after three months of treatment. Patients with sub-item 4 presented a slower improvement. However, it remained constant over time and finally resulted in the most relevant reduction in the MIDAS scores.

One possible explanation for the improvement in “control” could be that as the severity and frequency of migraines decrease, some of the compulsion to abuse drugs tends to subside. Several patients have transitioned from a CM + MOH condition to an episodic form without drug abuse. Patients with MOH often exhibit a lack of control over impulsivity, with a tendency for compulsive substance abuse to alleviate anxiety about an impending migraine attack. With the benefits brought about by OBT-A therapy, patients experienced fewer migraine attacks and improved compulsive behaviours. In this context, reducing the sub-item “controlling” corresponding to the controlling compulsion may reflect this improvement.

Nonetheless, the absence of a control group made it impossible to directly compare our patients with those receiving treatments other than OBT-A. However, our data, along with the existing literature, strongly suggest the importance of this therapy in decreasing the frequency of migraine attacks.

### Limitations

This study had several limitations, most notably the lack of a control group. This weakness made it impossible to compare the results observed with OBT-A with other treatments. We will take this limit into account for future developments of this study. The small sample size represented another limitation: although we examined the OC profile in detail, the small number of patients limits the generalisability of our results. However, as this was a pilot study, we are expanding our sample to obtain stronger and more generalisable results. Another limitation is represented by the short time window: we only examined the enrolled subjects for 9 months; however, with the encouraging results obtained in the present investigation, we are also extending the observation time.

This type of study has some possible bias, for example, a significant difference between males and females, or the absence of some age groups. To overcome these biases, it will be necessary to enlarge the sample of patients examined, or include this type of examination in a multicentre study. We aim to consider these possibilities in the future to expand our pilot study.

## 4. Conclusions

The preliminary observations collected in this pilot study suggest a need for further exploration of the psychiatric traits associated with migraines, such as OCD, which are often inadequately assessed. Moreover, our results highlight an opportunity to expand the understanding of all potential therapeutic possibilities of OBT-A. Based on our findings, this treatment not only appears to be effective in controlling pain, but is also capable of improving other aspects that characterise the negative impact of OCD on affected patients.

## 5. Materials and Methods

### 5.1. Study Population

We enrolled all patients with CM + MOH followed by the Headache Centre, Neurological Clinic, Marche Polytechnic University, who underwent OBT-A therapy over a 9-month period.

The diagnosis of CM + MOH was made according to IHS guidelines [36]. Each patient underwent an initial clinical evaluation, during which we collected the medical history and performed a general and neurological examination. Each patient was required to have had a brain imaging examination (CT or MRI) in the six months prior to enrolment to rule out any pathological findings, particularly brain neoplasms or ischemic lesions.

Inclusion criteria were: (a) diagnosis of CM + MOH according to the IHS 2018 criteria [36]; (b) clinical indication for therapy with OBT-A according to international guidelines [37]; (c) no significant improvement in migraine severity after prophylaxis with one or more previous prophylactic drugs (selected from the classes of drugs indicated by the IHS guidelines [38]: beta-blockers, calcium channel blockers, antidepressants, and antiepileptics); (d) age >18 years; (e) discontinuation of all prophylactic medications after starting OBT-A therapy; and (f) normal neurological and general examination at baseline and at follow-up visits.

Exclusion criteria were: (a) current use of drugs acting on the central nervous system or mood stabilizers, also considering drugs used for migraine prophylaxis; (b) documented history of psychiatric illness; (c) irregular intake or intolerance to OBT-A therapy; and (d) brain imaging (CT or MRI) showing evidence of tumours, ischemic or haemorrhagic damage, or other significant brain injury.

We decided to exclude patients taking drugs acting on the central nervous system or mood stabilisers because the effect of medication could alter our questionnaire scores by reducing the presence or perception of symptoms in patients.

Patients were evaluated prospectively at the beginning of OBT-A therapy (T0) and then every three months for a total of three evaluations: T1 (after three months of treatment), T2 (after six months), and T3 (after nine months). For each evaluation, patients underwent a comprehensive medical history assessment that included the record of monthly migraine days (MMDs) and the number of monthly medications taken to manage acute attacks (migraine acute medication intake, MAMI). They also completed migraine burden questionnaires (MIDAS, HIT-6) and the OCI-R to evaluate the presence and severity of obsessive-compulsive traits. The OCI-R was analysed both as an overall score and for each sub-item. This battery of questionnaires was repeated for each patient assessment. Patients regularly maintained a headache diary to track the severity and frequency of their attacks.

Patients received OBT-A therapy at each visit as per the PREEMPT study protocol (155 I.U., 5 I.U. for 31 injection sites) [39]. In selected cases, following the “follow pain” mode, we increased the dosage to a maximum of 195 I.U. [40]. We administered three rounds of injections, one every three months, for a total of nine months of treatment.

All participants gave their written informed consent according to the Declaration of Helsinki. The study protocol was approved by the Ethics Committee of the Marche Region (CERM), Italy (protocol number 202266).

### 5.2. Statistical Analysis

We considered the following variables at enrolment (T0):

- Continuous: sex: age, MMD, MAMI, MIDAS score, HIT-6 score, global OCI-R, global OCI-R in percentiles, global control, global control in percentiles, global sorting, global sorting in percentiles, global washout, global washout in percentiles, global obsession, global obsession in percentiles, global hoarding, global hoarding in percentiles, global mental neutralisation, and mental neutralisation in percentiles.
- Dichotomous: gender.
- Categorical: type of drugs used to manage migraine attacks, OCI-R category, control category, order category, washout category, hoarding category, and mental neutralisation category.

At the follow-up times (T1, T2, T3), we considered the following variables:

- Continuous: MMDs, MAMI, MIDAS score, HIT-6 score, global OCI-R, global OCI-R in percentiles, global checking, global checking in percentiles, global ordering, global ordering in percentiles, global washing, global washing in percentiles, global obsessing, global obsessing in percentiles, global hoarding, global hoarding in percentiles, global mental neutralising, and mental neutralising in percentiles.
- Categorical: type of medication used for migraine management, OCI-R category, checking category, ordering category, washing category, hoarding category, and mental neutralising category.

All continuous variables were evaluated for normality using the Kolmogorov–Smirnov test. Those with a normal distribution were compared using the *t*-test for repeated measures (two levels) or the ANOVA test for repeated measures (multiple levels), and results were presented as the mean, standard deviation (SD), and range. Continuous variables exhibiting a non-normal distribution and categorical variables were compared using the Mann–Whitney U test (two levels) or the Friedman test for repeated measures (multiple levels), and the results were presented as the median and range. Categorical and dichotomous variables were analysed using the chi-square test, with the results presented as absolute numbers and percentages. The statistically significant differences in the ANOVA test for repeated measures were also assessed by the same model by correcting for age and sex, adopting type 2 or type 3 sum of squares.

All differences were deemed significant at a threshold of *p* < 0.05. Sample size and power analysis were performed using G*Power version 3.1.9.3 for macOS systems, while statistical analysis was executed with Jamovi version 2.3.28.0 for macOS systems.

### 5.3. Sample Size Analysis

For the between-effect factor of the ANOVA model for repeated measures, we considered an effect size (f) of 0.45, an alpha error of 0.05, two groups, four repeated measures, and a sample size of 30 subjects to achieve a statistical power of 85%. For the within-effect factor of the ANOVA model for repeated measures, we considered an effect size (f) of 0.3, an alpha error of 0.05, two groups, four repeated measures, and a sample of 26 subjects to achieve a statistical power of 95%.

## Figures and Tables

**Table 1:** Characteristics of the cohort at T0, T1, T2, and T3. Epidemiological data and principal migraine variables are indicated to better characterize the sample. We also explain all the OCI-R categories.

Cohort Characteristics				
**Age (years)** **Mean (SD); [range]**			47.8 (11.3); [20.0–66.0]	
**Sex** **Male** **Female**			7 (23.3%) 23 (76.7%)	
*Variables collected at T0, T1, T2, and T3*				
	**T0 (n = 30)**	**T1 (n = 25)**	**T2 (n = 18)**	**T3 (n = 15)**
**MMD (days)** **Mean (SD); [range]**	26.9 (3.8); [20.0–30.0]	16.7 (7.1); [4.0–30.0]	12.7 (5.7); [4.0–27.0]	9.3 (6.0); [3.0–25.0]
**MAMI (tablets)** **Mean (SD); [range]**	28.4 (6.5); [20.0–50.0]	17.2 (8.1); [4.0–40.0]	12.8 (6.5); [4.0–30.0]	9.5 (7.3); [3.0–30.0]
**Drugs for the acute attack** **Triptans** **NSAIDS**	18 (60.0%) 12 (40.0%)	16 (64.0%) 9 (36.0%)	10 (55.6%) 8 (44.4%)	14 (93.3%) 1 (6.7%)
**MIDAS score** **Mean (SD); [range]**	89.7 (12.7); [55.0–105.0]	42.8 (30.0); [2.0–90.0]	27.1 (18.4); [3.0–67.0]	16.1 (16.8); [0.0–60.0]
**HIT-6 score** **Mean (SD); [range]**	67.1 (3.6); [61.0–74.0]	62.1 (4.6); [50.0–71.0]	60.2 (4.0); [50.0–66.0]	56.7 (3.7); [53.0–66.0]
**Global OCI-R** **Mean (SD); [range]**	14.0 (9.4); [0.0–33.0]	11.0 (9.3); [0.0–35.0]	10.2 (8.9); [0.0–35.0]	10.1 (8.7); [0.0–35.0]
**Global OCI-R (percentiles)** **Mean (SD); [range]**	67.8 (23.3); [10.0–99.0]	59.0 (28.9); [10.0–99.0]	57.0 (26.1); [10.0–99.0]	57.1 (24.8); [10.0–99.0]
**OCI-R category** **1** **2** **3** **4**	22 (73.3%) -- -- 8 (26.7%)	17 (68.0%) 2 (8.0%) 3 (12.0%) 3 (12.0%)	15 (83.3%) -- 1 (5.6%) 2 (11.1%)	13 (86.7%) -- 1 (6.7%) 1 (6.7%)
**Global checking** **Median; [range]**	3.5 [0.0–9.0]	1 [0.0–11.0]	1.5 [0.0–8.0]	2 [0.0–8.0]
**Global checking (percentiles)** **Mean (SD); [range]**	69.6 (32.6); [10.0–99.0]	56.9 (35.1); [10.0–99.0]	51.5 (35.3); [10.0–98.0]	54.2 (33.4); [10.0–98.0]
**Checking categories** **1** **2** **3**	15 (50.0%) 12 (40.0%) 3 (10.0%)	17 (68.0%) 6 (24.0%) 2 (8.0%)	15 (83.3%) 2 (11.1%) 1 (5.6%)	13 (86.7%) 1 (6.7%) 1 (6.7%)
**Global ordering** **Median; [range]**	2 [0.0–9.0]	1 [0.0–9.0]	1.5 [0.0–9.0]	2 [0.0–7.0]
**Global ordering (percentiles)** **Mean (SD); [range]**	66.3 (31.1); [10.0–99.0]	50.1 (37.6); [10.0–99.0]	49.6 (37.8); [10.0–99.0]	59.0 (37.3); [10.0–97.0]
**Ordering categories** **1** **2** **3**	17 (56.7%) 9 (30.0%) 4 (13.3%)	17 (68.0%) 6 (24.0%) 2 (8.0%)	12 (66.7%) 5 (27.8%) 1 (5.6%)	8 (53.3%) 5 (33.3%) 2 (13.3%)
**Global washing** **Median; [range]**	0 [0.0–3.0]	0 [0.0–7.0]	0 [0.0–3.0]	0 [0.0–3.0]
**Global washing (percentiles)** **Mean (SD); [range]**	26.7 (26.5); [10.0–85.0]	20.3 (25.0); [10.0–98.0]	0.2 (0.7); [10.0–85.0]	24.3 (29.7); [10.0–85.0]
**Washing categories** **1** **2** **3**	28 (93.3%) 2 (6.7%) --	24 (96.0%) -- 1 (4.0%)	16.9 (20.7) 1 (5.6%) --	14 (93.3%) 1 (6.7%) --
**Global obsessing** **Median; [range]**	2 [0.0–10.0]	2 [0.0–10.0]	2 [0.0–10.0]	2 [0.0–10.0]
**Global obsessing (percentiles)** **Mean (SD); [range]**	64.1 (32.4); [10.0–99.0]	61.0 (34.4); [10.0–99.0]	59.0 (33.7); [10.0–99.0]	65.1 (25.3); [10.0–99.0]
**Obsessing categories** **1** **2** **3**	19 (63.3%) 7 (23.3%) 4 (13.3%)	15 (60.0%) 8 (32.0%) 2 (8.0%)	13 (72.2%) 3 (16.7%) 2 (11.1%)	12 (80.0%) 2 (13.3%) 1 (6.7%)
**Global hoarding** **Median; [range]**	2 [0.0–8.0]	1 [0.0–5.0]	1.5 [0.0–5.0]	1 [0.0–4.0]
**Global hoarding (percentiles)** **Mean (SD); [range]**	60.1 (28.9); [10.0–98.0]	51.6 (28.1); [10.0–90.0]	48.9 (31.2); [10.0–90.0]	42.3 (30.0); [10.0–85.0]
**Hoarding categories** **1** **2**	28 (93.3%) 2 (6.7%)	25 (100.0%) --	18 (100.0%) --	15 (100.0%) --
**Mental neutralizing** **Median; [range]**	0 [0.0–7.0]	0 [0.0–8.0]	0 [0.0–8.0]	0 [0.0–8.0]
**Mental neutralizing (percentiles)** **Mean (SD); [range]**	43.7 (42.1); [10.0–99.0]	36.5 (39.6); [10.0–99.0]	46.2 (41.8); [10.0–99.0]	38.1 (41.1); [10.0–99.0]
**Mental neutralizing categories** **1** **2**	21 (70.0%) 9 (30.0%)	21 (84.0%) 4 (16.0%)	16 (88.9%) 2 (11.1%)	14 (93.3%) 1 (6.7%)

Legend: HIT-6 = headache impact test; MAMI = migraine acute medication intake; MIDAS = migraine disability assessment score; MMDs = monthly migraine days; SD = standard deviation; OCI-R = Obsessive-Compulsive Inventory-Revised.

**Table 2:** Estimated marginal means of the ANOVA model between T0 and T3 for the MIDAS score.

			95% CI	
MIDAS Score	Mean	SE	Lower	Upper
**T0**	92.3	2.29	87.41	97.3
**T1**	44.2	7.66	27.71	60.8
**T2**	29.0	4.33	19.60	38.3
**T3**	17.5	4.62	7.51	27.5

Legend: MIDAS = migraine disability assessment score; CI = confidence interval; SE = standard error of the mean.

**Table 3:** Estimated marginal means of the ANOVA model between T0 and T3 for the MIDAS score categories according to the OCIR category item.

				95% CI	
OCI-R (T0)	MIDAS Score	Mean	SE	Lower	Upper
**1**	**T0**	95.5	2.64	89.80	101.2
	**T1**	26.1	8.84	7.00	45.2
	**T2**	18.5	5.00	7.70	29.3
	**T3**	13.4	5.34	1.86	24.9
**4**	**T0**	89.2	3.73	81.14	97.3
	**T1**	62.4	12.50	35.39	89.4
	**T2**	39.4	7.07	24.12	54.7
	**T3**	21.6	7.55	5.28	37.9

Legend: CI = confidence interval; OCI-R = Obsessive-Compulsive Inventory-Revised; SE = standard error of the mean.

**Table 4:** Estimated marginal means of the ANOVA model between T0 and T3 for the MIDAS score categories according to the checking category item.

				95% CI	
Checking (T0)	MIDAS Score	Mean	SE	Lower	Upper
**1**	**T0**	95.6	3.10	88.873	102.4
	**T1**	19.6	8.87	0.306	38.9
	**T2**	19.9	5.33	8.258	31.5
	**T3**	16.4	6.16	2.963	29.8
**2**	**T0**	90.0	3.58	82.204	97.8
	**T1**	54.3	10.24	32.026	76.6
	**T2**	26.0	6.16	12.586	39.4
	**T3**	18.5	7.11	3.014	34.0
**3**	**T0**	96.0	8.76	76.903	115.1
	**T1**	90.0	25.08	35.359	144.6
	**T2**	67.0	15.08	34.144	99.9
	**T3**	0.00	17.41	−37.934	37.9

Legend: MIDAS = migraine disability assessment score; CI = confidence interval; SE = standard error of the mean.

## Data Availability

The datasets presented in this article are not readily available because of privacy issues. Requests to access the datasets should be directed to the corresponding author under reasonable request.

## Abbreviations

The following abbreviations are used in this manuscript:

HIT-6	Headache impact test
CM	Chronic migraine
MOH	Medication overuse headache
OCD	Obsessive-compulsive disorder
OBT-A	Onabotulinum toxin A
OCI-R	Obsessive-Compulsive Inventory-Revised
OC	Obsessive-compulsive
MAMI	Migraine acute medication intake
MIDAS	Migraine disability assessment score
MMDs	Monthly migraine days
SD	Standard deviation
CERM	Ethics Committee of the Marche Region
